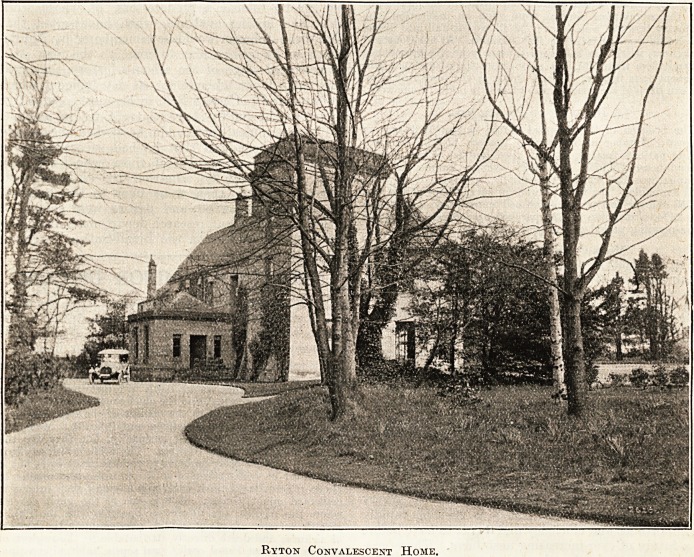# A "Live Hospital"

**Published:** 1923-11

**Authors:** 


### A "LIVE" HOSPITAL.

### NEWCASTLE ROYAL VICTORIA INFIRMARY.

THE Innes-Hopkins Memorial Convalescent Home of the Royal Victoria Infirmary at Newcastle-on-Tyne was formally opened by the donor on October 6. It has been given by Lieut.-Col. C. H. Innes-Hopkins, his wife and surviving family in memory of his two sons who were killed in the war. This important addition to the facilities of the infirmary is a fine house with grounds beautifully situated in an elevated position in the picturesque Tyneside village of Ryton, and is by no means the only benefaction which the donor has made to the infirmary. The estimated weekly cost per patient will be £2, which will be reduced to £1 10s. if there are twenty patients in residence, and to £1 5s. if the home contains its full complement of thirty. Mr. Charles Irwin, a member of the Innes-Hopkins Memorial Home Committee, declares that he can run the home for a year if £1,000 is subscribed.

Ryton Convalescent Home.

### Good Finance.

The Governors of the Infirmary are spending £3,000 upon extending the existing accommodation in the Pathological Department, and so the buildings are practically ready for occupation. An appeal is being issued for money with which to purchase Insulin. The Infirmary has already spent a considerable amount on this drug, but the Governors are anxious that the funds should be provided by public appeal rather than by drawing upon the already over-burdened finances of the institution. The financial statement for the eight months ending August 31 shows that the total income from all sources amounted to £59,478, and the total expenditure to £59,202, being an excess of income over expenditure of £276. This position is to a great extent due to payments received from approved societies towards the maintenance cost of their members, which amounted to nearly £3,700. The bank overdraft, however, stands at £6,100, to which the expenditure for the building and equipment of the pathological extensions will have to be added. The income is £2,798 more than for the corresponding period of last year, while the expenditure has been reduced by £3,531.

### How It is Done.

Commenting upon these figures, Mr. S. Dunstan, the House Governor and Secretary, writes to us :—

This position may, to a great extent, be attributed to the fact that in Newcastle the hospital is the pet charity of the district and that everyone is more or less interested. We have 76 representatives on the House Committee, and during last year 180 House Committee and Sub-Committee meetings were held. Outside the hospital we have also 77 local committees set up, which are all doing good work. There are between five and six thousand Governors on our books, any of whom is allowed to visit the institution between the hours of 2 and 6 p.m., and during the ordinary visiting hours each is allowed to bring one friend. The hospital is visited on an average twice weekly by parties representing the various trades and works in the district. In addition, two Governors are appointed each week by the Committee to visit any department of the hospital and to report thereon in a book kept for the purpose. Each week the patients in the hospital are visited by between six and seven thousand people.

### Crowded Courts of Governors.

I firmly believe that if the general public were more enlightened and could be brought more into touch with the great work which is daily going on inside our hospital walls and the advantages derived from such an institution, the voluntary hospital would never be allowed to fall into disuse or have its work curtailed by lack of funds. At our Courts of Governors, which are held four times a year, the attendance is rarely less than 300, but more often the attendance will represent four figures. This, I think, is the best indication one can have of the general interest that is taken in our work.

### A Decrease of £28 per Bed.

The position of this hospital at the end of 1922 was as follows: Number of In-patients treated, 11,876; number of Out-patients treated, 93,406; total number of Out-patient attendances, 276,079. The daily average number of beds occupied was 525, which represents 98.3 per cent. of the total beds available. The total number of surgical operations was 11,755 and the cost per occupied bed on ordinary expenditure was £174, a decrease of £28 per bed as compared with the corresponding period of the preceding year.

### More Co-Ordination Needed.

I am strongly of opinion that there is a great lack of co-ordination and co-operation between outside bodies, such as those responsible for the management and working of cottage hospitals and other bodies which are charged more or less with the responsibility of looking after the sick and lame poor, and if all the available beds in the district were made use of, our waiting list, which at present stands at 1,222, could be very materially reduced and the community in general much better served. At the present time this Hospital has on its books sixteen patients who have been in residence, on an average, over four months.

## Figures and Tables

**Figure f1:**